# Text mining brain imaging reports

**DOI:** 10.1186/s13326-019-0211-7

**Published:** 2019-11-12

**Authors:** Beatrice Alex, Claire Grover, Richard Tobin, Cathie Sudlow, Grant Mair, William Whiteley

**Affiliations:** 1 School of Informatics, University of Edinburgh, Informatics Forum, 10 Crichton Street, Edinburgh, UK; 2 Edinburgh Futures Institute, School of Literatures, Languages and Cultures, University of Edinburgh, 50 George Square, Edinburgh, UK; 3 The Alan Turing Institute, The British Library, 96 Euston Road, London, UK; 4 Centre for Medical Informatics, University of Edinburgh, 9 Little France Road, Edinburgh, UK; 5 Centre for Clinical Brain Sciences, University of Edinburgh, Chancellor’s Building, 49 Little France Crescent, Edinburgh, UK

**Keywords:** Text mining, Electronic healthcare records, Neuroimaging reports, Stroke classification

## Abstract

**Background:**

With the improvements to text mining technology and the availability of large unstructured Electronic Healthcare Records (EHR) datasets, it is now possible to extract structured information from raw text contained within EHR at reasonably high accuracy. We describe a text mining system for classifying radiologists’ reports of CT and MRI brain scans, assigning labels indicating occurrence and type of stroke, as well as other observations. Our system, the Edinburgh Information Extraction for Radiology reports (EdIE-R) system, which we describe here, was developed and tested on a collection of radiology reports.The work reported in this paper is based on 1168 radiology reports from the Edinburgh Stroke Study (ESS), a hospital-based register of stroke and transient ischaemic attack patients. We manually created annotations for this data in parallel with developing the rule-based EdIE-R system to identify phenotype information related to stroke in radiology reports. This process was iterative and domain expert feedback was considered at each iteration to adapt and tune the EdIE-R text mining system which identifies entities, negation and relations between entities in each report and determines report-level labels (phenotypes).

**Results:**

The inter-annotator agreement (IAA) for all types of annotations is high at 96.96 for entities, 96.46 for negation, 95.84 for relations and 94.02 for labels. The equivalent system scores on the blind test set are equally high at 95.49 for entities, 94.41 for negation, 98.27 for relations and 96.39 for labels for the first annotator and 96.86, 96.01, 96.53 and 92.61, respectively for the second annotator.

**Conclusion:**

Automated reading of such EHR data at such high levels of accuracies opens up avenues for population health monitoring and audit, and can provide a resource for epidemiological studies. We are in the process of validating EdIE-R in separate larger cohorts in NHS England and Scotland. The manually annotated ESS corpus will be available for research purposes on application.

## Background

The goal of the EdIE-R system [1] is to label each report with an indication of what the radiologist was able to observe in the scan image, for example, *small vessel disease*, *ischaemic stroke* etc. Like most other systems for extracting information from electronic healthcare records, we use text mining techniques to identify the relevant parts of the report which can then be used as a basis for predicting the document-level labels.

**Fig. 1:**
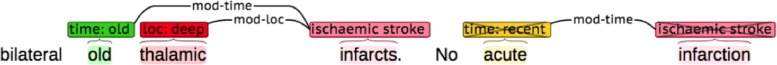
Example of entity, relation and negation mark-up

Text mining systems typically apply Named Entity Recognition (NER), Relation Extraction (RE) and negation detection. NER is used to identify words or phrases that are ‘entities’ relevant to the text mining task and RE links entities when they are related in some relevant way. Negation detection identifies contexts where the author is stating that entities or relations do not exist. For example, Fig. 1 shows different types of annotations: two ischaemic stroke entities, *infarcts* and *infarction*, two temporal modifiers, *old* and *acute*, and a location modifier, *thalamic*. The first ischaemic stroke entity enters into two relations, one with a temporal modifier and one with a location modifier, while the second ischaemic stroke entity is in a relation with a temporal modifier. These latter two entities are marked as negative (crossed out) because they are in the scope of the negative word *No*. Annotations such as these are output by the text mining system and are then used as the basis for the assignment of labels to the reports.

In order to develop NER and RE components, decisions need to be made about which entities and which relations the system should identify. These decisions are best made through dialogue between the domain experts, who know what information they would ideally like to access, and text mining experts, who can judge which pieces of information can be identified with sufficient accuracy. In addition, manually annotated subsets of the data are needed to train and develop the components as well as to evaluate their performance.

In building EdIE-R, we used the process of annotation as a means to focus the radiologist/text miner dialogue at the same time as developing the prototype system. We used an agile development methodology where iterations of system development were interleaved with annotation iterations. After initial scoping, automatic annotations from the system were presented to the domain experts for correction using the BRAT annotation tool [2]. The system and manual annotations were compared and disagreements were resolved either by adjusting the manual annotation or by improving the system. We iterated over the process a number of times with both system and manual annotation improving in each cycle. This method has several advantages. First, it allows both teams to work simultaneously, unlike methods where all the annotation is done in advance of system development. Second, discussion of the system and manual disagreements allows the text miners to come to a much clearer understanding of the meaning of the domain language and the domain specialists to understand the limitations of the technology. Through negotiation, several changes to the annotation scheme were made during the iterative process. Third, doing annotation as correction tends to reduce insignificant differences between manual and system annotation.

## Related work

Named entity recognition is a well-established task in NLP. The CoNLL shared-task evaluations [3] established benchmarks for NER evaluation and prompted research into supervised machine learning methods for NER, for example, the Stanford NER tagger [4]. Rule-based techniques are also still used for NER: see e.g. the ANNIE NER tagger which is part of GATE [5]. Relation extraction is often included as a subtask in text mining applications [6] with approaches to it ranging from rule-based through supervised to unsupervised machine learning.

Text mining technology for the biomedical domain has been a subject of research for two decades with several community initiatives to provide data and a forum for shared tasks, such as BioCreative [7] and BioNLP [8]. Both of these organised shared tasks in NE and RE: see [9, 10] for our contributions. More recently the shared task approach has been used for electronic health records (EHRs) by the LOUHI workshops, e.g. LOUHI’17 [11] or LOUHI’18 [12]. There are many individual studies applying information extraction to EHRs, see [13] for a review of some of these. Negation detection has been recognised as an important step, particularly in medical text mining, with the NegEx algorithm [14] being frequently used.

**Fig. 2:**
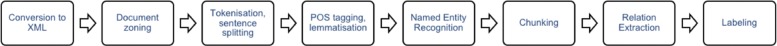
EdIE-R processing pipeline

Several researchers have applied NLP and text mining approaches to radiology reports. Pons et al. (2016) provide a useful systematic review of NLP in radiology [15]. They include 67 different studies which they group according to 5 distinct purposes, namely diagnostic surveillance, cohort building for epidemiological studies, query-based case retrieval, quality assessment of radiologic practice, and clinical support services. Conditions targeted by the systems are various and include appendicitis, pneumonia, renal cysts, pulmonary embolism, liver conditions and general metastases, to name but a few. Across all these application areas the NLP systems surveyed tend to have the same broad structure where a flow diagram showing the individual components looks much like our diagram of the EdIE-R system shown in Fig. 2 below.

Two recent studies by Hassanpour and Langlotz (2016) and by Cornegruta et al. (2016) describe machine learning methods for entity recognition from radiology reports [16, 17]. Hassanpour and Langlotz [16] tested two existing feature-based machine learning classifiers for this task. Their annotation scheme contains four broad types of named entities (*Anatomy*, *Anatomy modifier*, *Observation* and *Observation modifier*) as well as strings expressing *Uncertainty*. They used NegEx to identify negation in the text as a feature feeding into their models. The machine learning classifier both result in an average F1-score of 85% for 10-fold cross-validation on a data set containing 150 manually annotated radiology reports from three different institutions.

Cornegruta et al. [17] describe work on analysing a large corpus of historical chest X-ray reports. Their system described is interestingly similar to ours in the way the report text is annotated with named entity and negation mark-up although the entity list (Body Location, Descriptor, Clinical Finding, Medical Device) is both smaller and more complex in that disjoint entities are permitted. No relation extraction is performed but negation mark-up is included. The NER method uses a bidirectional LSTM (BiLSTM) neural network architecture, which is contrasted with a baseline system which uses string matching look-up against RadLex [18] and Medical Subject Headings (MeSH) [19] concepts combined with parsing, plus NegEx for negation detection. The BiLSTM NER tagger significantly outperforms the baseline but it is worth noting that, in general, rule-based and machine learning approaches attain similar levels of performance on NER if the rule-based system uses more sophisticated techniques than string matching, as ours does.

There has also been some work on summarising radiology reports. Most recently, Zhang et al. [20] proposed a state-of-the-art neural network-based approach to summarisation of radiology impressions. An impression is the “Conclusion” section of a radiology report summarised by the radiologist after dictating or writing down their findings presented in the image. Automating this step is an extremely useful task that can save radiologists a lot of effort and time. Two different radiology reports describing similar symptoms and conditions, however, are not guaranteed to result in the same summary text. The output of summarisation therefore does not lend itself well for large-scale data analysis in the same way as classification of symptoms and conditions does, for example, for identifying patients with the same findings for epidemiological studies.

With a specific focus on stroke, Flynn et al. (2010) [21] developed a system for analysis of brain scan radiology reports from Tayside, Scotland, i.e. EHR reports which are very similar to the those in the ESS data set [22]. Their aim was to improve on the coding of the reports which were frequently given generic ‘stroke’ codes even when a more precise code could be determined by looking at the report. Their method used a keyword matching step looking for affirmative or negative uses of key words from a stroke lexicon. They report results which were acceptably accurate in identifying ischaemic stroke (94.7% positive predictive value (precision)) on a dataset of 150 reports manually classified as ischaemic stroke. Their method performed less reliably in identifying intracerebral haemorrhage (76.7% positive predictive value) on a dataset of 150 reports manually classified as intracerebral haemorrhage. The paper does not report sensitivity (recall) scores as the data only contains positive examples of either type.

To the best of our knowledge, EdIE-R is the first system that performs named entity extraction, negated entity detection, relation extraction and document level labelling with the goal to classify radiology report with types of stroke, tumours and other information. The extracted entities (positive or negative) and relations are all used to do the final classification (labelling) step. The information captured in and about the reports include a comprehensive set of entities and labels. We provide a detailed evaluation of EdIE-R for all the steps it is designed to perform using standard natural language processing evaluation metrics, including precision, recall and F1-score. Compared to the previous study [21] we therefore test on an unseen test set of random radiology reports which contain positive and negative examples of the information EdIE-R is designed to extract and label.

## Method

### Annotation scheme

**Fig. 3:**
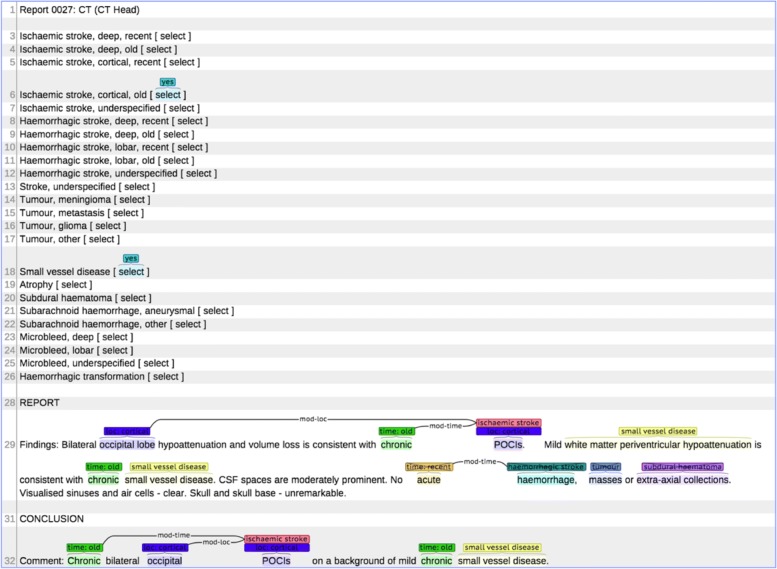
An annotated report

There are four aspects to the annotation of brain scan reports in our data: entities, relations, negation mark-up, and labels. These are all illustrated in Fig. 3, a screen grab of an annotated report loaded into the BRAT tool. As shown, each report is preceded by a list of all possible labels but only those that have been marked as selected are labels for the report. Entities, relations and negation have been annotated within the textual body of the report.

Entities are of two types, observations or modifiers. The full set of observation entities are: ischaemic stroke, haemorrhagic stroke, stroke (unknown type), tumour:meningioma, tumour:metastasis, tumour:glioma, tumour, subdural haematoma, small vessel disease, atrophy, microbleed, subarachnoid haemorrhage and haemorrhagic transformation. The four modifier entities, which are used to identify location (deep vs. cortical/lobar) and recency (old vs. recent) of an observation, are loc:deep, loc:cortical, time:old, time:recent.

Relations link a subset of observation entities, namely stroke and microbleed entities, with modifier entities. Strokes may be associated with both a location and a time, while microbleeds are associated only with location. Some words or phrases, such as POCI (Posterior Circulation Infarct) in Fig. 2, carry both observation and modifier meaning and in these cases nested entities are used. Here there is a mod-loc relation between the loc:cortical entity and the ischaemic stroke entity but we do not require this to be made explicit in the annotation since the nesting implies it.

There is a close relationship between the entity and relation names and the labels. For example, the label Ischaemic stroke, cortical, old has been chosen and this clearly relates to the two occurrences of an ischaemic stroke entity in a relation with both a loc:cortical and a time:old modifier. The annotators are instructed not to select labels unless there is explicit linguistic evidence to support the choice. Occasionally they will be able to infer labels from implicit information but they are asked not to annotate these cases as the aim is to model linguistically explicit information not human expertise.

Proper identification of negation and its scope is essential to achieving high accuracy. We model negation in the annotation as an attribute on entities, which is visualized in BRAT as a crossing out. Wherever the text contains negation scoping over entities, the annotators must add the negative attribute. The negative example in Fig. 2, *No acute haemorrhage, masses or extra-axial collections*, is a clear and simple case but syntactically more complex cases occur, e.g. cases where the negation marker is distant from the entities within its scope. There are cases where the radiologist is unable to positively identify or exclude an observation, as for example in *a small focus of acute infarct cannot be completely excluded*. The annotators are asked to mark these cases as negative, as only clearly positive observations should contribute to the labels assigned to the reports.

### The EdIE-R system

EdIE-R is a rule-based text mining system which we developed in tandem with manual data annotation in the form of correction of the system output. The presentation of the data in the BRAT tool, as illustrated in Fig. 2, is the view that the annotators see, but this is a format that has been derived from the data structure which the system manipulates and outputs, which is an XML data structure. We have developed the system’s text analysis components using the LT-XML2 programs, which are the core of our XML rule-based text mining software [23]. Our most recent software release, the Edinburgh Geoparser [24], contains all of our general-purpose components, such as the tokeniser, NER tagger and chunker, which we have adapted to the brain scan report domain in EdIE-R.

**Fig. 4:**
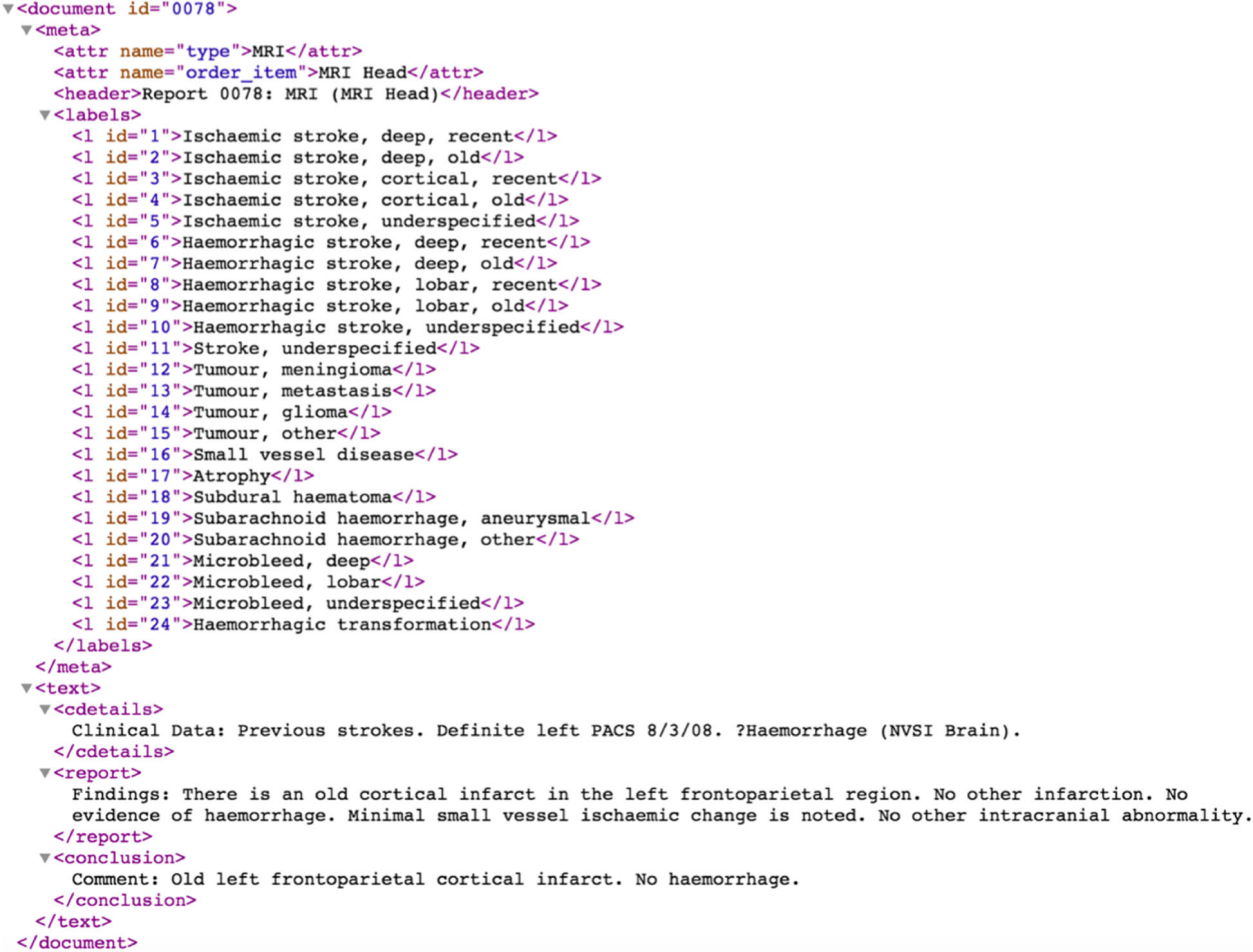
XML format after document zoning

As shown in Fig. 3, the EdIE-R system has a pipeline architecture. Scan reports are converted from their original format into an initial XML format and subsequent components incrementally add annotations to the XML structure, with each stage making computations over the annotations of previous stages. The document zoning step segments the reports into sections including clinical details, the report itself and the radiologist’s conclusion. It also adds metadata which includes all of the possible labels that can be assigned; by the final stage of the pipeline an attribute on each label indicates whether that label has been selected. An example of a report in XML after document zoning is shown in Fig. 4. We combine NER and label mark-up in this way so that manual annotation of all levels of analysis can be done at the same time.

Subsequent steps of the pipeline do linguistic processing. The tokeniser splits textual content into paragraphs, sentences and word tokens, with punctuation characters also treated as tokens. The C&C POS tagger [25] labels each word with its syntactic category. The default C&C model has been trained on modern U.S. newspaper text and although it performs well on most text types, it is not wholly suitable for the medical text in our reports. For this reason, we also use a model trained on the Genia biomedical corpus [26]. After running the POS tagger with each of the models we apply a correction stage to moderate disagreements between them. After POS tagging, we apply the morpha lemmatiser [27] to analyse inflected nouns and verbs and compute their lemma (morphological stem). The output of POS tagging and lemmatization is stored in attribute values on word token elements.

**Fig. 5:**
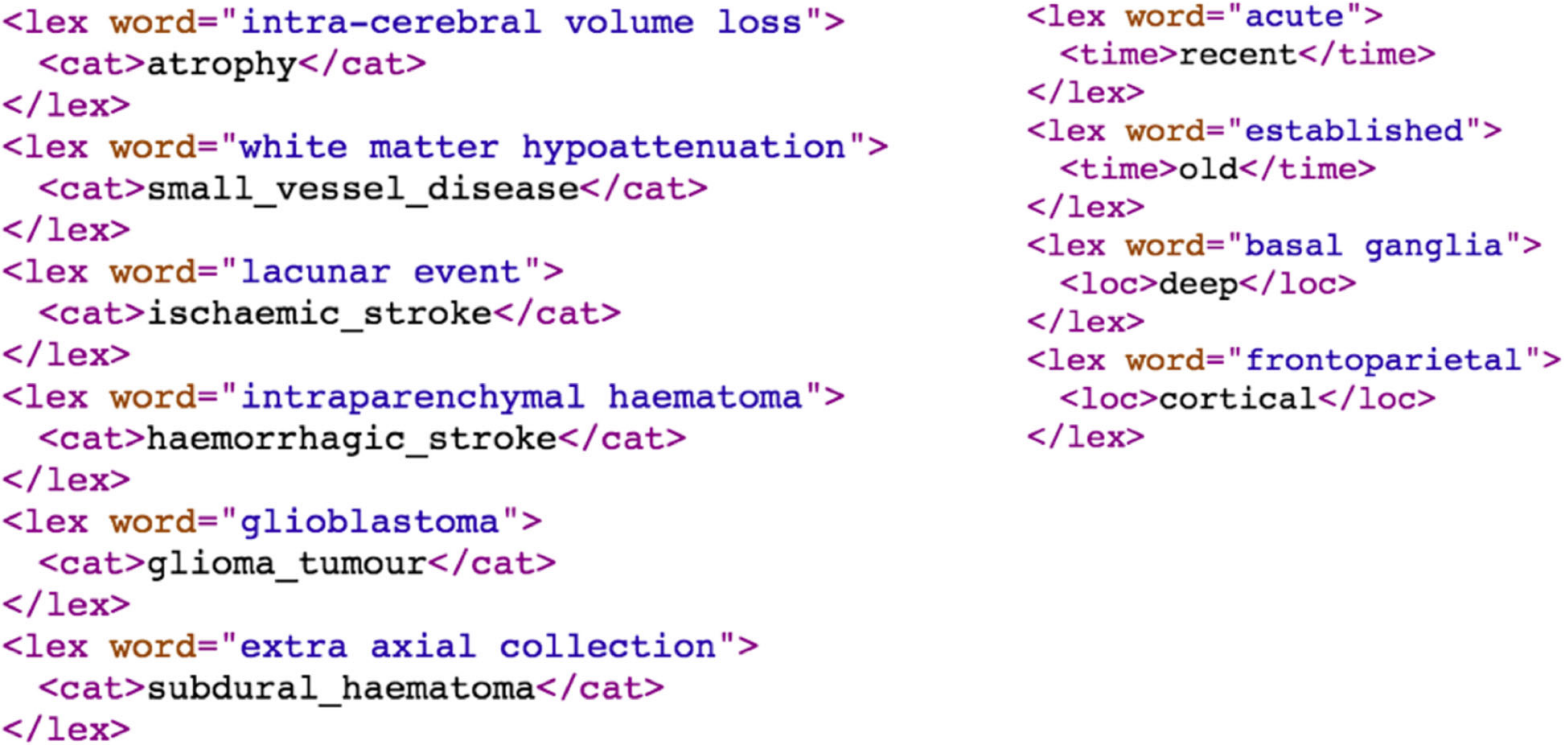
Example lexical entries

The fifth step in the pipeline is the NER component, which incorporates lexical lookup. From examples in the development set we manually curated two lexicons, one for observations (e.g. the atrophy entity *inter-cerebral volume loss* and the ischaemic stroke entity *lacunar event*) and one for modifiers (e.g. the time:old entities *old*, *previous* and *established*), e.g. see Fig. 5. The process of lexical lookup results in the addition of further attributes to the word tokens of matching words and phrases. The lexicons are applied one after the other, first the observations lexicon and then the modifiers, so that some words or phrases can be marked as both observation and modifier to achieve the nested entity mark-up described above.

The next stage of processing performs a shallow syntactic analysis using our chunker [28] to segment sentences into phrases or word groups, i.e. syntactic structures headed by nouns (noun groups), verbs (verb groups) etc. The purpose of doing this is to create a useful data structure for dealing with nested entities and coordinations of entities as well as to define the scope of negation markers in terms of structure rather than just word sequences. At this stage complex negative noun groups such as *No acute haemorrhage, masses or extra-axial collections* have an appropriate structure to allow information from the negative article *No* to be propagated through the group so that all three observation entities (*haemorrhage*, *masses*, *extra-axial collections*) are marked as negative.

**Fig. 6:**
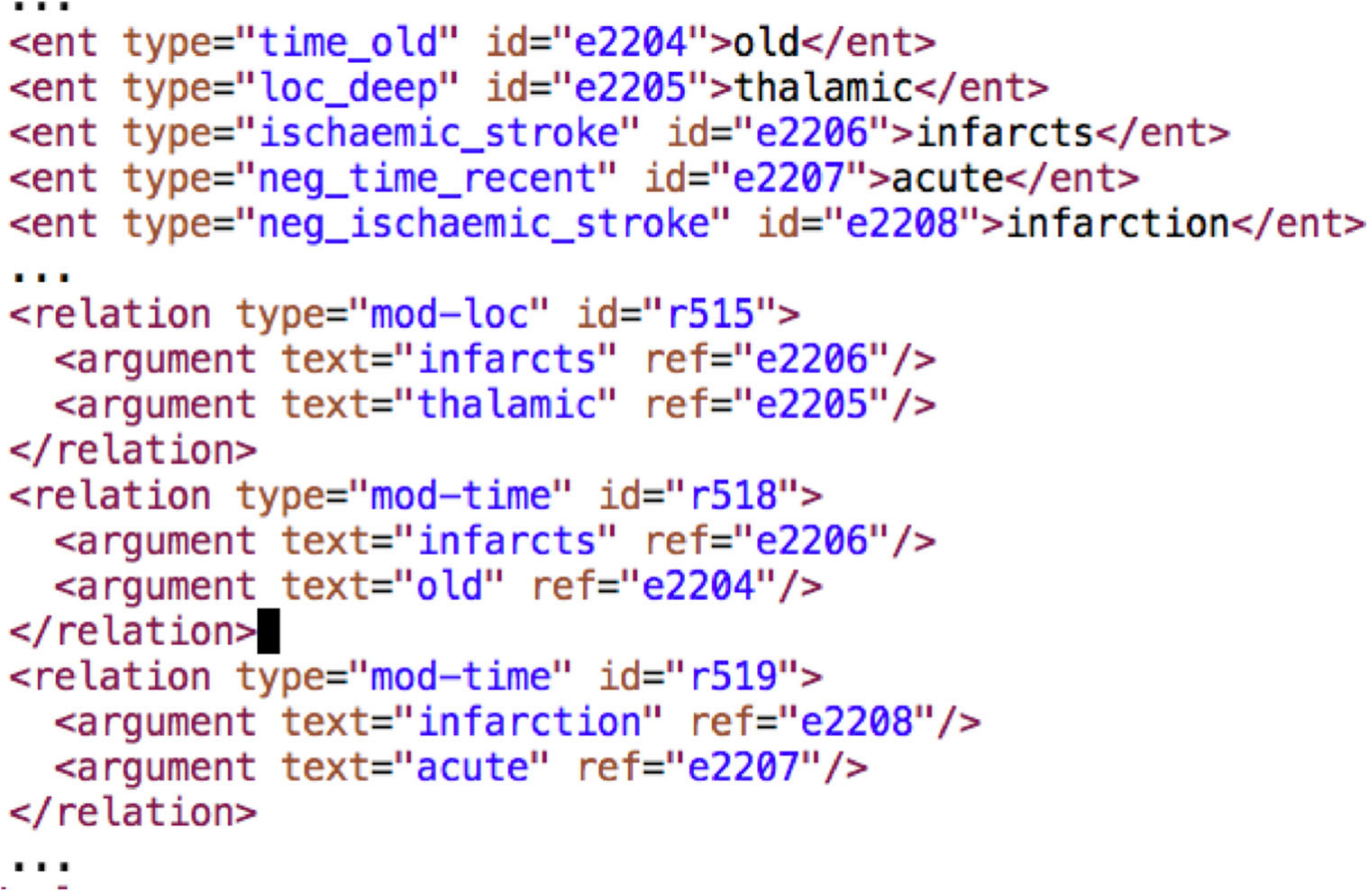
XML representation of entities and relations in Fig. 1

Relation Extraction is the final stage of the text mining part of the system. In this component some pairs of entities are linked in relations held as structures in standoff XML mark-up as illustrated in Fig. 6. There are two possible relations, location and time, which hold between stroke entities (ischaemic, haemorrhagic or unknown type) and modifiers. In addition, a *microbleed* entity can be in a relation with a location modifier.

Negation arising from the verb particle *not*, for example in *Very acute infarction may not be visible on CT*, is handled as part of the relation extraction module because rules linking *not* with the entities it scopes over are similar to the other relation rules. The result, however, is not an explicit relation but an attribute on the negated entities (*acute* and *infarction*, in this case). This is the same format as for noun group negation detected during chunking.

The final labelling step of the pipeline uses the information from the previous steps to compute which labels should be associated with a record. Because the mark-up coming from the text mining is very detailed, the labeling rules can be fairly simple. For example, to choose the Small vessel disease label the rules need only to check that there is a non-negative small vessel disease entity in either the report or conclusions part of the report. To choose the label Ischaemic stroke, cortical, recent there needs to be a non-negative ischaemic stroke entity which is in a location relation (mod:loc) with a cortical location entity (loc:cortical) and in a time relation (mod:time) with a time:recent entity. There are a few added complexities to these rules, for example, a deep ischaemic stroke which is not in an explicit relationship with a time modifier is assumed to be old.

### Evaluation

In order to evaluate system performance, we annotated development and test data as discussed in the “Annotation scheme” section. For this we used 1168 reports from the Edinburgh Stroke Study (ESS) [22]. We reserved the first 500 reports as the development set and the remainder as the test set. ESS contains MRI, CT and Doppler Ultrasound reports but we used only the CT and MRI reports. We also discarded a few reports which contained non-brain results, e.g. combined brain and neck, chest, or abdomen scans. In total the annotated development set contains 322 CT and 42 MRI reports. We have annotated a random subset of the test set containing 238 CT and 28 MRI reports.

Manual annotation of the development data was accomplished in six tranches, where annotation was correction of the system output. The system was modified and improved between the tranches. Table 1 provides information on the sizes of the data subsets. The first three tranches were doubly annotated by the radiology experts so that IAA could be monitored. For these three tranches only, disagreements between the annotators were reconciled to produce an agreed gold standard. The remaining development data was singly annotated. The test data was doubly annotated in three tranches but not reconciled. Table 2 provides details of the annotators and annotations in all the data sets.

**Table 1:** The annotated ESS data sets

	Reports	Of which CT	Of which MRI	Sentences	Words
Development					
dev1	18	18	0	158	1651
dev2	25	16	9	231	2671
dev3	80	78	2	888	6833
dev4	82	74	8	833	6935
dev5	82	69	13	965	8,061
dev6	77	67	10	762	6078
Total	364	322	42	3837	32,229
Test					
test1	89	74	15	969	7,919
test2	92	82	10	996	8,226
test3	85	82	3	890	6697
Total	266	238	28	2855	22,842

**Table 2:** Annotations in the data sets

	Annotated by	Positive entities	Negative entities	Relations	Labels
dev1	Both: reconciled	197	85	68	46
dev2	Both: reconciled	242	116	85	62
dev3	Both: reconciled	670	324	230	192
dev4	Annotator 1	600	284	195	167
dev5	Annotator 2	708	302	212	174
dev6	Annotator 1	524	280	169	151
Total		2941	1391	959	792
test1	Annotator 1	605	291	203	159
test2	Annotator 1	786	337	278	192
test3	Annotator 1	572	333	206	167
Total		1963	961	687	518
test1	Annotator 2	614	304	220	160
test2	Annotator 2	792	361	281	199
test3	Annotator 2	574	355	200	176
Total		1980	1020	701	535

## Results

Following standard practice we measure both IAA and system performance using precision, recall and F1. Note that IAA represents an upper bound for system performance as an automatic method would not be expected to out-perform human capabilities. The overall results for IAA on the test data are shown in Table 3. Note that IAA measures for relations are only computed for those relations where the two annotators agree on both entities linked by the relation. Overall the IAA results are very high which indicates that the annotation task is well-defined.

**Table 3:** Inter-annotator agreement on the test data

	Precision	Recall	F1
Entities			
test1	96.41	98.77	97.57
test2	95.84	98.40	97.10
test3	94.94	97.46	96.18
Total	95.73	98.22	96.96
Negation			
test1	95.90	98.19	97.03
test2	95.07	97.70	96.36
test3	94.73	97.29	96.00
Total	95.22	97.72	96.46
Relations			
test1	92.99	98.03	95.44
test2	97.47	97.47	97.47
test3	96.39	91.67	93.97
Total	95.77	95.91	95.84
Labels			
test1	92.50	93.08	92.79
test2	90.95	94.27	92.58
test3	94.32	99.40	96.79
Total	92.52	95.56	94.02

Tables 4, 5 and 6 provide a more detailed breakdown of the IAA results per type on the entities, relations and labels across the three test sets. The majority of lower IAA scores for entity types are for low frequency ones, for example subarachnoid haemorrhage. This pattern is mirrored in the IAA scores for labels, for example for Haemorrhagic transformation and Microbleed. However, since these types are very infrequent their low IAA scores do not have a serious effect on the overall figures.

**Table 4:** IAA precision, recall and F1 for entities including numbers of true positives (TP), false positives (FP) and false negatives (FN)

Type	TP	FP	FN	Precision	Recall	F1
Entities						
ischaemic stroke	453	9	2	98.05	99.56	98.80
haemorrhagic stroke	264	20	3	92.96	98.88	95.83
stroke (unknown type)	25	0	1	100.00	96.15	98.04
tumour:meningioma	8	0	0	100.00	100.00	100.00
tumour:metastasis	12	0	0	100.00	100.00	100.00
tumour	165	2	1	98.80	99.40	99.10
subdural haematoma	109	32	0	77.30	100.00	87.20
small vessel disease	269	15	7	94.72	97.46	96.07
atrophy	147	14	6	91.30	96.08	93.63
microhaemorrhage	10	0	0	100.00	100.00	100.00
subarachnoid haemorrhage	9	3	1	75.00	90.00	81.82
haemorrhagic transformation	2	2	0	50.00	100.00	66.67
time:old	314	9	7	97.21	97.82	97.52
time:recent	354	0	0	100.00	100.00	100.00
loc:cortical	410	5	2	98.80	99.51	99.15
loc:deep	321	17	22	94.97	93.59	94.27
TOTAL	2872	128	52	95.73	98.22	96.96

**Table 5:** IAA precision, recall and F1 for relations including numbers of TPs, FPs and FNs

Type	TP	FP	FN	Precision	Recall	F1
Relations						
mod-loc	235	17	25	93.25	90.38	91.80
mod-time	421	12	3	97.23	99.29	98.25
TOTAL	656	29	28	95.77	95.91	95.84

**Table 6:** IAA precision, recall and F1 for labels including numbers of TPs, FPs and FNs

Type	TP	FP	FN	Precision	Recall	F1
Labels						
Ischaemic stroke, deep, recent	4	0	0	100	100	100
Ischaemic stroke, deep, old	81	4	4	95.29	95.29	95.29
Ischaemic stroke, cortical, recent	13	3	1	81.25	92.86	86.67
Ischaemic stroke, cortical, old	58	6	3	90.62	95.08	92.8
Ischaemic stroke, underspecified	6	6	6	50	50	50
Haemorrhagic stroke, deep, recent	2	1	0	66.67	100	80
Haemorrhagic stroke, deep, old	4	0	0	100	100	100
Haemorrhagic stroke, lobar, recent	4	0	0	100	100	100
Haemorrhagic stroke, lobar, old	3	0	0	100	100	100
Haemorrhagic stroke, underspecified	9	0	1	100	90	94.74
Stroke, underspecified	14	1	1	93.33	93.33	93.33
Tumour, meningioma	4	0	0	100	100	100
Tumour, metastasis	0	0	0	-	-	-
Tumour, glioma	0	0	0	-	-	-
Tumour, other	2	3	1	40	66.67	50
Small vessel disease	158	3	1	98.14	99.37	98.75
Atrophy	119	9	3	92.97	97.54	95.2
Subdural haematoma	6	0	0	100	100	100
Subarachnoid haemorrhage, aneurysmal	0	0	0	-	-	-
Subarachnoid haemorrhage, other	5	2	1	71.43	83.33	76.92
Microbleed, deep	1	1	0	50	100	66.67
Microbleed, lobar	1	0	0	100	100	100
Microbleed, underspecified	0	0	1	NaN	0	NaN
Haemorrhagic transformation	1	1	0	50	100	66.67
TOTAL	495	40	23	92.52	95.56	94.02

Table 7 shows evaluation results for the EdIE-R system on the two annotators’ versions of the test set. For labels and relations, the system agrees more with Annotator 1 than with Annotator 2, while the pattern is reversed for entities and negation. We would expect system scores to be lower than IAA (see final column), which is the case for entities and negation for Annotator 1, and for all but relations for Annotator 2. We speculate that these differences indicate that Annotator 1 focused more on entity mark-up and spotted and corrected more system entity errors while Annotator 2 focused more on the labels and made more corrections there. To improve the accuracy of the evaluation we would ideally arbitrate the annotators’ disagreements and produce a consensus test set. Nevertheless, the overall evaluation results are reassuringly high, indicating that this method of labelling radiology reports is highly effective.

**Table 7:** Evaluation of the system on the two annotators’ test sets. We reproduce IAA from Table 3 for comparison

	Precision	Recall	F1	IAA F1
Entities				
Annotator 1 test set	94.63	96.37	95.49	96.96
Annotator 2 test set	97.21	96.50	96.86	
Negation				
Annotator 1 test set	93.54	95.30	94.41	96.46
Annotator 2 test set	96.35	95.66	96.01	
Relations				
Annotator 1 test set	97.32	99.24	98.27	95.84
Annotator 2 test set	95.47	97.61	96.53	
Labels				
Annotator 1 test set	94.94	97.88	96.39	94.02
Annotator 2 test set	92.70	92.52	92.61	

In Table 8 we provide a breakdown of system performance for the labelling task as compared with Annotator 2. This shows the comparative frequency of the different labels. Small vessel disease and Atrophy are the most frequent and the system performs well on both. The presence of these labels boosts the total precision, recall and F1 into the low 90s. With the exception of Ischaemic stroke, deep, old and Haemorrhagic stroke, deep, recent, performance is generally slightly lower for both Ischaemic and Haemorrhagic stroke labels than the total entity score. The comparative frequency of these labels (Ischaemic more frequent than Haemorrhagic) does not appear to make a difference in Table 8, but it may be that the number of Haemorrhagic stroke instances is too low for the sample to be representative. Similarly, other labels are so infrequent that their results may not be interpretable and it would be useful to acquire and annotate more data to improve the robustness of the evaluation results.

**Table 8:** Detailed evaluation of system labelling compared to Annotator 2 showing numbers of true positives (TP), false positives (FP) and false negatives (FP), as well as precision, recall and F1

Type	TP	FP	FN	Precision	Recall	F1
Ischaemic stroke, deep, recent	4	1	0	80.00	100.00	88.89
Ischaemic stroke, deep, old	81	5	4	94.19	95.29	94.74
Ischaemic stroke, cortical, recent	14	1	2	93.33	87.50	90.32
Ischaemic stroke, cortical, old	56	5	8	91.80	87.50	89.60
Ischaemic stroke, underspecified	6	8	6	42.86	50.00	46.15
Haemorrhagic stroke, deep, recent	3	0	0	100.00	100.00	100.00
Haemorrhagic stroke, deep, old	4	1	0	80.00	100.00	88.89
Haemorrhagic stroke, lobar, recent	4	1	0	80.00	100.00	88.89
Haemorrhagic stroke, lobar, old	3	1	0	75.00	100.00	85.71
Haemorrhagic stroke, underspecified	9	3	0	75.00	100.00	85.71
Stroke, underspecified	13	1	2	92.86	86.67	89.66
Tumour, meningioma	4	1	0	80.00	100.00	88.89
Tumour, metastasis	0	3	0	0.00	-	-
Tumour, glioma	0	0	0	-	-	-
Tumour, other	4	2	1	66.67	80.00	72.73
Small vessel disease	158	0	3	100.00	98.14	99.06
Atrophy	120	3	8	97.56	93.75	95.62
Subdural haematoma	5	0	1	100.00	83.33	90.91
Subarachnoid haemorrhage, aneurysmal	0	0	0	-	-	-
Subarachnoid haemorrhage, other	4	1	3	80.00	57.14	66.67
Microbleed, deep	1	0	1	100.00	50.00	66.67
Microbleed, lobar	1	0	0	100.00	100.00	100.00
Microbleed, underspecified	0	2	0	0.00	-	-
Haemorrhagic transformation	1	0	1	100.00	50.00	66.67
TOTAL	495	39	40	92.70	92.52	92.61

## Conclusion

We have described the development and evaluation of the EdIE-R system on brain imaging radiology reports from the Edinburgh Stroke Study. The evaluation results are encouraging and the system is sufficiently accurate that we believe it can be used for its intended purpose of data provision for epidemiological studies. To that end, we are currently testing and revising the system on a dataset of over 150,000 routine brain scans from NHS Tayside collected between 1994 and 2015. We are also in the process of evaluating whether the system can reliably identify cases of intracerebral haemorrhage in patients in Greater Manchester.

The evaluation of EdIE-R against these larger datasets will show how robust it is against new data. The disadvantage of a rule-based system such as EdIE-R is that it takes time to write the rules. However, we found that with the help of the domain expert input we were able to get a first prototype running fairly quickly. For a small dataset such as ESS, we found this to work very well as we did not have any training data available at the start to test machine learning methods. Now that we have the annotated data ready we are evaluating machine learning approaches in parallel to investigate if we can obtain better results using them.

## Data Availability

The annotated ESS corpus that we have created as part of this project has much potential value as a resource for developing text mining algorithms. This data will be available on application to Prof. Cathie Sudlow (email: Cathie.Sudlow AT ed.ac.uk) to bona fide researchers with a clear analysis plan, in line with the Wellcome Trust policy on data-sharing (https://wellcome.ac.uk/what-we-do/topics/data-sharing). We are in the process of creating a release of EdIE-R free for research purposes (https://www.ltg.ed.ac.uk/software/edie-r). For more information contact Dr. Beatrice Alex (email: balex AT ed.ac.uk).
